# Domain topology and domain switching kinetics in a hybrid improper ferroelectric

**DOI:** 10.1038/ncomms11602

**Published:** 2016-05-24

**Authors:** F. -T. Huang, F. Xue, B. Gao, L. H. Wang, X. Luo, W. Cai, X. -Z. Lu, J. M. Rondinelli, L. Q. Chen, S. -W. Cheong

**Affiliations:** 1 Rutgers Center for Emergent Materials, Department of Physics and Astronomy, Rutgers University, Piscataway, New Jersey 08854, USA; 2 Department of Materials Science and Engineering, Pennsylvania State University, University Park, Pennsylvania 16802, USA; 3 Laboratory for Pohang Emergent Materials, Max Plank POSTECH Center for Complex Phase Materials, Pohang University of Science and Technology, Pohang 790-784, Korea; 4 Department of Materials Science and Engineering, Northwestern University, Evanston, Illinois 60208, USA

## Abstract

Charged polar interfaces such as charged ferroelectric walls or heterostructured interfaces of ZnO/(Zn,Mg)O and LaAlO_3_/SrTiO_3_, across which the normal component of electric polarization changes suddenly, can host large two-dimensional conduction. Charged ferroelectric walls, which are energetically unfavourable in general, were found to be mysteriously abundant in hybrid improper ferroelectric (Ca,Sr)_3_Ti_2_O_7_ crystals. From the exploration of antiphase boundaries in bilayer-perovskites, here we discover that each of four polarization-direction states is degenerate with two antiphase domains, and these eight structural variants form a Z_4_ × Z_2_ domain structure with Z_3_ vortices and five distinct types of domain walls, whose topology is directly relevant to the presence of abundant charged walls. We also discover a zipper-like nature of antiphase boundaries, which are the reversible creation/annihilation centres of pairs of two types of ferroelectric walls (and also Z_3_-vortex pairs) in 90° and 180° polarization switching. Our results demonstrate the unexpectedly rich nature of hybrid improper ferroelectricity.

## Abstract (web summary)

Charged ferroelectric domain walls show promise for two-dimensional conduction, but their abundance within (Ca,Sr)_3_Ti_2_O_7_ crystals is poorly understood. Here, Huang *et al*. discover topology related domain structures in such materials, which reveal the rich nature of hybrid improper ferroelectricity.

## Graphical abstract

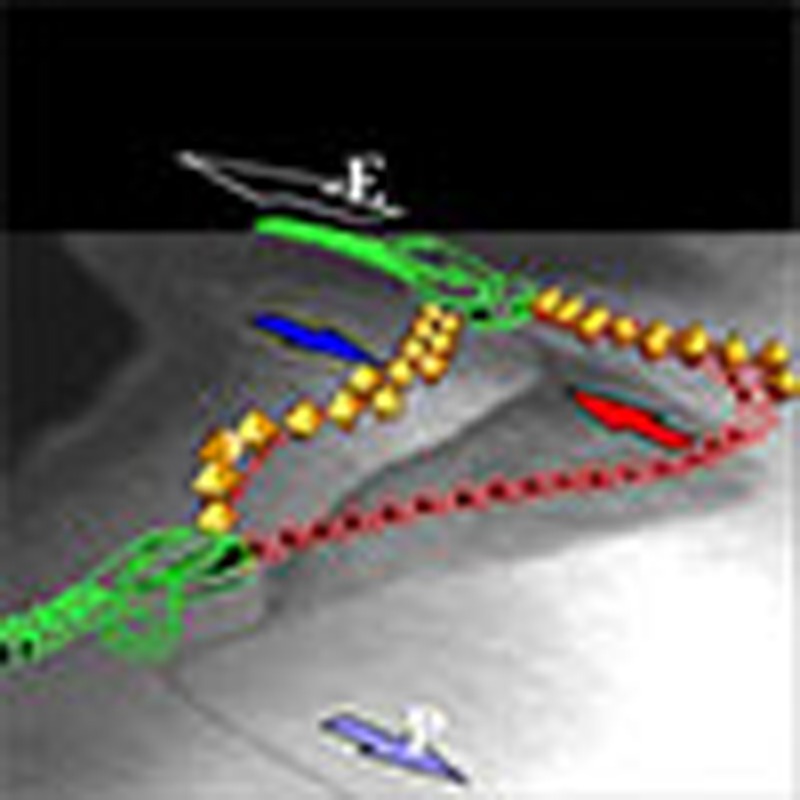

Over the last decade, two-dimensional (2D) conduction in heterostructured interfaces with polar discontinuity^1^,^2^ or compositionally homogeneous charged interfaces such as charged ferroelectric domain walls (FE DWs)^3^,^4^,^5^,^6^,^7^,^8^,^9^,^10^ has attracted enormous attention for emergent phenomena and new material functionalities. However, undesirable chemical/structural complexity such as ionic diffusion, oxygen vacancies or structural/strain variations near the interface results in equivocal interpretation of the origin of 2D conduction^11^,^12^,^13^. In parallel, significant efforts have been launched to investigate the 2D conduction at charged FE DWs, which are well defined at the atomic scale. The conducting FE DWs were observed in chemically homogeneous thin films, for example, Pb[Zr_x_Ti_1-x_]O_3_ (ref. 14), BiFeO_3_ (ref. 10) and Bi_0.9_La_0.1_FeO_3_/SrRuO_3_ heterostructure^9^ and bulk single crystals, for example, LiNbO_3_ (ref. 4), BaTiO_3_ (ref. 5) and Er(Ho)MnO_3_ (refs 6, 7). These conducting FE DWs are intrinsically unstable because of large energy cost^8^,^15^, often pinned by chemical defects^4^ and sporadically^5^ and artificially created with external voltage^9^. However, charged domain walls (DWs), some of which are highly conducting, were found to be abundant in the hybrid improper FE (Ca,Sr)_3_Ti_2_O_7_ (ref. 3).

Ordering phase transitions in condensed matter can be accompanied by directional variants and antiphase boundaries (APBs). Directional-variants result in domains with different directional order parameters. The role of APBs^16^,^17^ on materials functionalities has been well recognized^18^,^19^,^20^,^21^,^22^. In particular, the discovery of strong interaction or interlocking nature of APBs with ferroic orders in diverse functional materials opens new grounds for material research. Examples include the ferromagnetic coupling in Heusler alloys^23^, the reduced spin polarization in half-metal magnetites^24^, the duality nature of DWs and topological defects in hexagonal manganites^25^, FE APBs in a nonpolar matrix^16^ and conducting and ferromagnetic walls of antiferromagnetic domains in pyrochlore iridates^26^.

Hybrid improper ferroelectricity (HIF), a phenomenon involving polarization induced by a hybridization of two non-polar lattice instabilities, offers great promise towards the realization of room-temperature multiferroism^27^,^28^,^29^,^30^,^31^. The key idea is to design new materials in which ferroelectricity and (anti)ferromagnetism can be coupled by the same lattice instability, therefore providing an indirect but strong coupling between polarization and magnetism^27^,^28^,^29^,^31^,^32^. Examples of compounds with HIF include the double-layered Ruddlesden-Popper perovskites with the chemical formula of A_3_B_2_O_7_ (Fig. 1, A^2+^=alkali metal; B^4+^=transition metal)^3^,^27^,^32^. Unexpectedly, charged FE DWs, some of which are highly conducting, were also found to be mysteriously abundant in the recently discovered Ruddlesden-Popper-type HIF (Ca,Sr)_3_Ti_2_O_7_ crystals^3^.

To unveil the origin of these abundant charged FE DWs, we have explored the complete connectivity of DWs in Ca_2.55_Sr_0.45_Ti_2_O_7_ (CSTO; FE Tc≈790 K) and Ca_3_Mn_1.9_Ti_0.1_O_7_ (CMTO; FE Tc≈360 K) single crystals with in-plane polarization along the pseudo-tetragonal [110] directions^3^,^30^,^33^, particularly with mapping of APBs using transmission electron microscopy (TEM). Note that APBs are invisible in piezoresponse force microscopy (PFM)^34^, which is usually a good method to map out FE domain configurations. Phase-field simulations^35^ were also conducted to understand the origin of domain configurations in CMTO and CSTO. Our results reveal that the formation of a unique Z_4_ × Z_2_ domain topology with Z_3_ vortices is responsible for the presence of abundant charged FE DWs in CSTO. In addition, we have also investigated the kinetics associated with polarization switching in CSTO, understanding of which is crucial for developing precise control of conducting FE walls.

## Results

### Z_4_ × Z_2_ domain structure with eight different states

**Figure 1:**
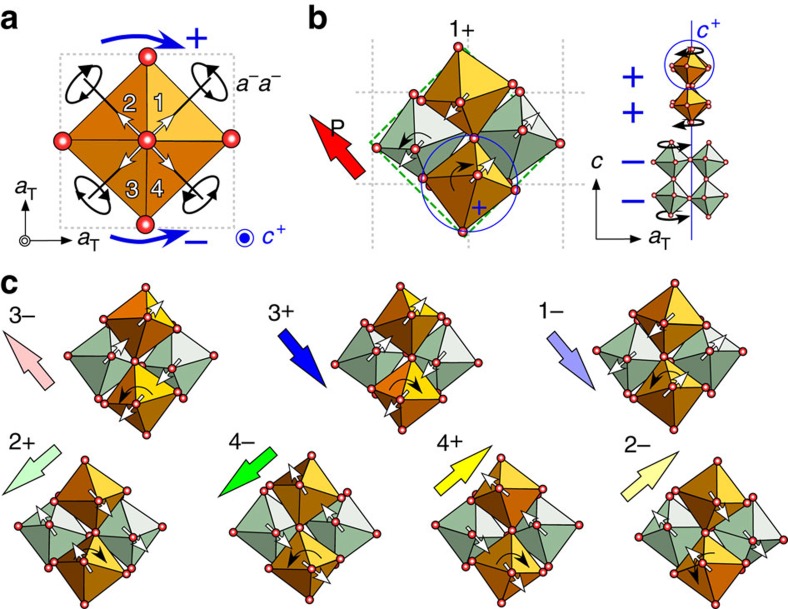
Structural illustrations of the eight ferroelectric domains in A_3_B_2_O_7_. (**a**) An in-plane BO_6_ octahedral constituent with tilting along the <110>_T_ directions and rotation around the [001]_T_ direction. Plus (+) and minus (−) represent clockwise and anticlockwise rotations. White arrows indicate the displaced directions (1^st^ to 4^th^ quadrants) of the apical oxygen of the BO_6_ octahedron due to octahedral tilting. The red spheres represents O ions. (**b**) The 1+ domain state. The red arrow indicates the polarization direction. The grey dotted lines depict the basic tetragonal framework constructed by B-site ions and the green dashed rectangle depicts the orthorhombic cell. The cross-sectional structure includes two bi-layers formed by orange and light green corner-sharing BO_6_ octahedra. Each domain state can be unambiguously identified by naming the distortions of a given octahedron (blue-circled) as the five adjacent octahedra in each bi-layer are constrained to tilt and to rotate in opposite senses, and also the overall crystallographic symmetry (*A2_1_am*) determines the distortions in the adjacent bi-layers. (**c**) The in-plane structural models of the 1−, 2±, 3± and 4± domain states. Switching of the octahedral tilting pattern is involved between two domain states in, for example, 1+ versus 3+, 1− versus 3−, 2+ versus 4+ and 2− versus 4−, and switching of the octahedral rotation pattern is required between 1+ versus 1−, 2+ versus 2−, 3+ versus 3− and 4− versus 4+. Antiphase domain relations can be found between two domain states in, for example, 1+ versus 3−, 1− versus 3+, 2+ versus 4− and 2− versus 4+. The corresponding polarization direction in each domain state can be derived readily from our nomenclature; for example, the 1+ domain state accompanies a polarization towards the 2nd quadrant—starting from the 1st quadrant and rotating clockwise (+) to the 4th quadrant that results in the nearest A-site cation (and polarization) being displaced in the opposite direction to the 2^nd^ quadrant. Note that the net in-plane dipole moment (Γ_5_^−^) is caused by this A-site-cation displacement.

**Figure 2:**
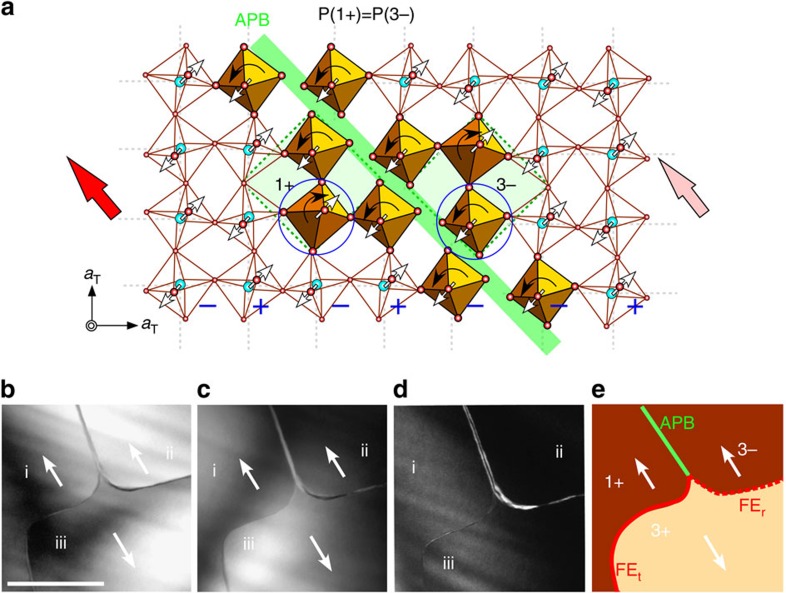
Antiphase boundary (APB) in a CSTO crystal. (**a**) The local distortions near an [110]_T_-oriented APB (green line) between the 1+ and 3− states, which are identical in polarization direction but differ in structure with respect to rotation (black curved arrows) and tilting (white arrows) by 180°. (**b**) A *ab*-plane DF-TEM image taken using superlattice **g**_**1**_^**+**^=3/2(1, 1, 0)_T_ spot. (**c**) A DF-TEM image taken using superlattice **g**_**1**_^−^=3/2(−1, −1, 0)_T_ spot. A reversed contrast in **b**,**c** demonstrates the characteristic of 180°-type FE domains. (**d**) A DF-TEM image taken using **g**_**1**_^−^ spot at a large tilting angle to tune contrast by enhancing excitation error. A clear boundary interference fringe can then be observed between domains ii and iii, implying an inclined nature and a strong strain gradient expected in rotation-driven FE_r_ DWs. (**e**) The schematic domain configuration obtained from **b**–**d** demonstrates a typical Z_3_ vortex pattern within a FA domain, composed of three 180°-FE domains and three DWs: FE_r_ (red-dotted), FE_t_ (red-solid) DWs and APB (green-solid). White arrows denote the polarization directions in FE domains. Scale bar, 500 nm.

Figure 1a shows two characteristic lattice modes in A_3_B_2_O_7_: First, the BO_6_ octahedral in-phase rotations are either clockwise (the sign of the rotation is +) or anticlockwise (−) about a [001]_T_ direction (denoted as *a*^0^*a*^0^*c*^+^ in the Glazer notation^36^ or the X_2_^+^ mode), and second, the BO_6_ octahedral tilting occurs about two <110>_T_ axes, that is, apical oxygen-motions displace towards the 1st to 4th quadrants (denoted as *a*^−^*a*^−^*c*^0^ or the X_3_^−^ mode) with respect to the high-symmetry tetragonal *I4/mmm* (T, space group #139) structure. Below the phase transition, the X_3_^−^ mode adopts one of the four tilts (1–4) accompanying the X_2_^+^ mode with + or − rotations into a combined distortion pattern of *a*^−^*a*^−^*c*^+^ having eight degenerate states, which we label as 1±, 2±, 3± and 4± (the complete structures are shown in Fig. 1b,c). Figure 1b shows the 1+ state projected along the [001]_T_ and [010]_T_ directions. For example, the apical oxygen of the blue-circled octahedron moves towards the 1^st^ quadrant (white arrow) with a clockwise rotation (black curved arrow) to form a 1+ state. A trilinear coupling among the X_2_^+^ mode, X_3_^−^ mode and the polar Γ_5_^−^ mode (A-site displacement) yields four FE polarizations parallel or antiparallel to the two <110>_T_ tilting axes^3^,^27^. We emphasize that each polarization direction is associated with two degenerate states, for example, the 1+ and 3− polarizations point in the same direction (red and light-red arrows in Fig. 1b,c), a consequence of both nonpolar order parameters (X_3_^−^ and X_2_^+^) changing signs. These four polarization directional variants with twofold degeneracy form the basis of the Z_4_ × Z_2_ domain structures in the HIF A_3_B_2_O_7_. Figure 2a shows a structural model of a APB (green line) between the 1+ and 3− states, at which the orthorhombic unit cells labelled by green dotted lines illustrate the discontinuation of octahedral tilting (white arrows) and rotation (+ and −) at the wall. APBs might exist in A_3_B_2_O_7_, but behave hidden in the PFM images as the two domains give the same piezo-response^3^.

### Z_3_ vortices in the Z_4_ × Z_2_ domain structures of (Ca,Sr)_3_Ti_2_O_7_

Polarized optical microscope images on CSTO and CMTO crystals both clearly exhibit orthorhombic twins, that is, orthorhombically distorted ferroelastic (FA) domains. In addition, in-plane PFM studies show the intriguing FE domains comprising abundant meandering head-to-head and tail-to-tail charged DWs^3^ (Supplementary Fig. 1). Compared with PFM, dark-field TEM (DF-TEM) under systematic controlled diffraction conditions allows us to light up domains induced directly by local structural deformations^37^. Figure 2b–d shows a series of DF-TEM images taken along the [001]_T_ direction using superlattice peaks **g**_**1**_^±^=±3/2(1, 1, 0)_T_ parallel to the polar axis within a single FA domain. Three domains (i–iii) in Fig. 2b–d, in which domains i and ii reveal the same domain contrast but opposite to domain iii in contrast, show the existence of antiphase domains i and ii, and an APB between them. Three FE domains including two antiphase domains merging at one vertex point are well illustrated in Fig. 2b–d. As a sense of rotation along the merging three domains is defined in the phase space (see below), their intersection can be called a Z_3_ vortex. Note that the relative polar ±a_orth_ directions can be identified from the related electron diffraction patterns but the absolute polarization direction cannot be. Thus, once the polarization direction is chosen for one domain, then the polarization directions in other domains can be fully assigned without ambiguity. Evidently, the existence of APBs remains unchanged even if the assignment of the polarization direction is reversed. Figure 2e depicts one possible assignment with 1+ and 3− antiphase domains. The APB between these 1+ and 3− domains accompanies the sign change of both rotation and tilting (Fig. 2a). The presence of an APB and a Z_3_ vortex also suggests the existence of pure rotation (*a*^0^*a*^0^*c*^+^)-driven DWs and pure tilting (*a*^−^*a*^−^*c*^0^)-driven DWs. We define a tilting-type FE_t_ DW (rotation-type FE_r_ DW) as the wall between adjacent FE domains having opposite *a*^−^*a*^−^*c*^0^ tilting (*a*^0^*a*^0^*c*^+^ rotation) but identical *a*^0^*a*^0^*c*^+^ rotation (*a*^−^*a*^−^*c*^0^ tilting). The structural details of FE_t_ and FE_r_ DWs are shown in Supplementary Figs 2 and 3. Note that the Z_3_ vortex in Fig. 2e consists of three distinct walls: FE_r_ (red-dotted), FE_t_ DWs (red-solid) and APB (green line).

**Figure 3:**
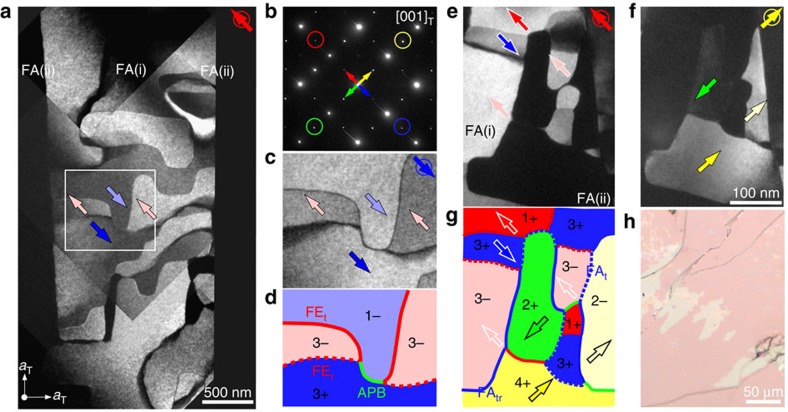
The Z_3_-vortex patterns in Ca_2.55_Sr_0.45_Ti_2_O_7_ and Ca_3_Mn_1.9_Ti_0.1_O_7_ crystals. (**a**) A 2.2  × 3.9 μm^2^ mosaic of DF-TEM images were taken using the superlattice **g**_**1**_^**+**^ spot (red-circled) of domain FA(i) in a CSTO crystal along [001]_T_. The coloured arrows represent polarization directions within the domain. (**b**) The electron diffraction pattern was taken by covering regions FA(i) and FA(ii) showing a 90°-crystallographic-twin relation. The red/blue circled spots were contributed from the orthorhombic distortions of the FA(i) region and the green/yellow ones were from the FA(ii) area. The colour-circled spot located in each DF image is the selected superlattice Bragg spot to light up the corresponding domains at a given orientation. (**c**) A DF-TEM image of the white rectangular box region was taken using the blue circled superlattice **g**_**1**_^−^ spot. (**d**) A proposed domain configuration of **c**. (**e**,**f**) DF-TEM images taken using (**e**) red circle and (**f**) yellow circle spots corresponding to superlattice **g**_**1**_^**+**^ spots in a CMTO crystal. A Z_3_-vortex network appears, with three DWs meeting at one point and with irregular shaped FE/FA domains. (**g**) A proposed domain configuration of **e**. Two types of FA DWs, FA_t_ (blue-dotted) and FA_tr_ (blue-solid), were identified. (**h**) Polarized optical image of a CMTO crystal showing irregular twin (that is, FA) domains in a hundreds micrometre scale.

Figure 3a shows a mosaic of DF-TEM images covering three FA (that is, orthorhombic twin; FA(i) and FA(ii)) regions in a CSTO crystal. In the DF-TEM image obtained using orthorhombic superlattice peaks **g**_**1**_^±^ contributed from the FA(i) domain marked by red and blue circles in Fig. 3b, the neighbouring FA(ii) regions exhibit a dark contrast (Fig. 3a). Aside from the major contrast between FA(i) and FA(ii), a self-organized Z_3_-vortex network is clearly visible within the FA(i) region. Figure 3c shows a reversed contrast within the white rectangular box taken using a **g**_**1**_^−^ spot (blue-circled) and the schematic (Fig. 3d) shows a pair of Z_3_ vortices is linked by an APB (green line). Boundaries between the 3− (pink) and 3+ (blue) domains form broad contrast walls, identified as FE_r_ DWs (red-dotted lines of Fig. 3d). By comparing the domain contrasts (Fig. 3c), wall features and the neighbouring FA domains obtained from our DF-TEM images, we completely assign all domain states and wall types appearing in Fig. 3a (see the Methods for details).

### Domain topology of Ca_3_(Mn,Ti)_2_O_7_

We also grew high-quality CMTO single crystals and confirmed the presence of polar domains in the same polar space group (*A2_1_am*) as Ca_3_Ti_2_O_7_ at 300 K. Figure 3h shows a polarized optical microscopy image of surprisingly irregular FA DWs on the cleaved (001)_T_ surface, distinct from the prototypical straight FA DWs (that is, orthorhombic twin walls) in CSTO. Our DF-TEM images (Fig. 3e,f) demonstrate consistently the presence of irregular twin patterns. The FA(i) domain is excited when the red-circled **g**_**1**_^+^ spot was used for imaging (Fig. 3e), and it turns to deep-dark contrast when the orthogonal yellow-circled spot was excited (Fig. 3f). Inside each FA domain, there exist 180°-type FE domains; for example, bright and grey contrast domains in Fig. 3e,f and Supplementary Fig. 4. The domain configuration shown in Fig. 3g displays the presence of Z_3_ vortices with three domains merging at the vortex cores, which exist within a FA domain, as well as at boundaries between FA domains. Thus, the configuration of Z_3_-vortex domains seems universal in the HIF A_3_B_2_O_7_, despite the existence of eight possible structural variants. Note that there exist two types of FA DWs: ferroelastic tilting DWs (FA_t_ DW) between states in the same rotation, for example, the 1+ and 2+ or 3− and 2− (blue-dotted lines in Fig. 3d and Supplementary Fig. 2), and FA tilting+rotation (FA_tr_) DWs between, for example, the 2+ and the 3− states (blue lines in Fig. 3d and Supplementary Fig. 2). A single tilting of either *a*^−^*a*^0^*c*^0^ or *a*^0^*a*^−^*c*^0^ type may occur at FA_t_ and FA_tr_ DWs (white arrows in Supplementary Fig. 2). FA_tr_ DWs seem naturally accompanied with a complete octahedral-rotation frustration at the walls, implying a higher energy of FA_tr_ DWs than that of FA_t_ DWs (Supplementary Fig. 3).

### Phase-field simulations

**Figure 4:**
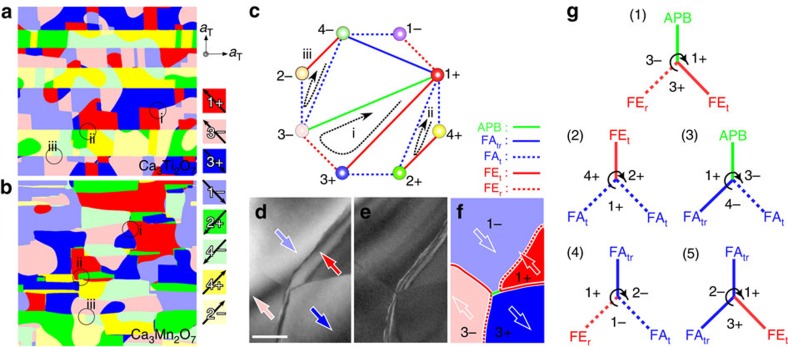
Z_4_ × Z_2_ domain with Z_3_ vortices in Ca_3_Ti_2_O_7_ and Ca_3_Mn_2_O_7_. (**a**,**b**) In-plane domain structures of Ca_3_Ti_2_O_7_ and Ca_3_Mn_2_O_7_ from phase-field simulations. The eight colours denote the eight domain variants as listed. Z_3_ vortices corresponding to loops i–iii are denoted by black circles in spite of different nature of FA domains in Ca_3_Ti_2_O_7_ and Ca_3_Mn_2_O_7_. (**c**) Schematic of the energy diagram in A_3_B_2_O_7_ compounds with eight vertices, representing eight domain variants. Each vertex is connected to seven edges that correspond to one of five types of DWs as shown in the right side. Loops i–iii depict possible vortex domains and domain walls (in fact, Z_3_ vortices). Note that non Z_3_-type vortex domains corresponding to loops connecting, for example, (1+, 3+, 3−, 1−, 1+) or (1+, 2+, 3+, 3−, 1+) have not been observed experimentally. (**d**–**f**) Experimental DF-TEM images demonstrate two Z_3_ vortices at a very short interval of 50 nm in a Ca_3_Ti_2_O_7_ crystal. Scale bar, 100 nm. (**d**) Image was taken under a Friedel's-pair-breaking condition to reveal 180°-type domain contrast. Polarization directions were shown by white arrows. (**e**) Image was taken under a larger tilting angle to reveal boundary interference fringes clearly. The width of bent fringes indicates a broad DW interrupting the connection of 1+ (red) and 3− (pink) domains. (**f**) Image shows a schematic of the corresponding domain configuration. (**g**) Five possible Z_3_-vortex configurations derived from the energy diagram. The indicated domain states are examples. Type 1, the only Z_3_-vortex configuration appearing within a single orthorhombic twin. Type 2, the most common Z_3_ vortex across the orthorhombic twin boundaries. Type 3, Z_3_ vortex accompanying 90° ferroelectric switching at APB. Type 4, Z_3_ vortex accompanying ferroelectric switching in the absence of APB. Type 5, the least favoured Z_3_ vortex with two high energy FA_tr_ walls.

We also employed the phase-field method (Methods and Tables 1, 2, 3)^35^ to investigate the domain structure of HIF A_3_B_2_O_7_, as shown in Fig. 4a,b. The details of the simulations are given in the Methods. The eight states are represented by different colours. Ca_3_Ti_2_O_7_ exhibits straight FA DWs (Fig. 4a), consistent with the experimental observation^3^ (Supplementary Fig. 1). Across the FA DWs, a dark colour tends to become another dark colour, for example, a red (1+) colour tends to change to a green (2+) colour, rather than to a light green (4−) colour (Fig. 4a, circle ii). Similarly, two light colours tend to be close with each other across the FA DWs (Fig. 4a, circle iii). These indicate that the rotation order parameter tends to be unchanged across the FA DWs^38^, which is consistent with the previous discussion on the low energy of FA_t_, compared with FA_tr_ DWs. Supplementary Fig. 3 shows the oxygen positions of the eight states relative to the tetragonal position. With the assumption that a wall going through a tetragonal-like state with zero polarization costs more energy, the energy hierarchy among the five DWs can be estimated to be FA_t_≤FE_r_≤FE_t_≤FA_tr_≤APB. The statistics of DW lengths obtained from phase-filed simulation results give a ratio of FE_r_:FA_t_:FE_t_:APB:FA_tr_=34:28:28:8:2 in Ca_3_Ti_2_O_7_ (Fig. 4a) and FA_t_:FE_r_:FA_tr_:FE_t_:APB=52:22:12:7:7 in Ca_3_Mn_2_O_7_ (Fig. 4b). A comparably large population of FE_t_, FE_r_ and FA_t_ walls, especially in Ca_3_Ti_2_O_7_, suggests that APB and FA_tr_ walls belong to the higher energy set than others. Experimentally, within a limited number of DWs that we have observed, CSTO exhibits an 82% population of the lower energy set (FE_r_, FE_t_ and FA_t_ walls), whereas CMTO shows a 64% population. The low population in CMTO is likely related with the existence of irregular FA domains in CMTO. Note that although an energy hierarchy may exist, we do observe experimentally and theoretically all five kinds of DWs.

The energy diagram for the HIF A_3_B_2_O_7_ such as Fig. 4c can be constructed with all eight states (vertices) and all five kinds of walls (edges connecting two vertices). Note that in Fig. 4c, only a small part of edges are shown, and each vertex is connected to all of the rest vertices through edges in the full energy diagram. The i, ii and iii loops in Fig. 4c correspond to Z_3_ vortices in the Fig. 4a,b, respectively. The presence of only Z_3_ vortices indicates that the energy difference among five types of DWs is not large, so the lowest-energy vortex defect is always Z_3_-type. Note that if, for example, APB is associated with much higher energy than others, then APB will be fully avoided, and Z_4_-type vortex defects such as 1+/3+/3−/1−/1+ can occur, but we observe only Z_3_-type vortices. In low-magnification TEM images, we sometimes observe vortices looking like Z_4_-type. However, all these likely Z_4_-type vortices turn out to be pairs of closely linked Z_3_ vortices with inclined broad DWs, as shown in Fig. 4d–f. The energy diagram (Fig. 4c) is, in fact, a hyper-tetrahedron in seven dimensions, which has eight vertices and only triangular faces. Each triangular face in this hyper-tetrahedron corresponds to a Z_3_ vortex. All possible configurations of Z_3_ vortex derived from the energy diagram (Fig. 4c) are shown in Fig. 4g and have been observed experimentally. The configuration of Z_3_ vortex domains seems universally adopted in HIF A_3_B_2_O_7_ compounds. Note that FA DWs in Ca_3_Ti_2_O_7_, nucleated from a high-temperature tetragonal phase (*I4/mmm*)^33^,^39^, tend to be straight, whereas FA DWs in Ca_3_Mn_2_O_7_, nucleated from the *Acaa* (space group #68) phase^30^ below ∼360 K, are irregular (Supplementary Fig 4). A phase-field simulation for the nucleation and growth of the FE *A2_1_am* domains from the *Acaa* matrix is shown in Supplementary Movie 1.

### Domain switching kinetics

**Figure 5:**
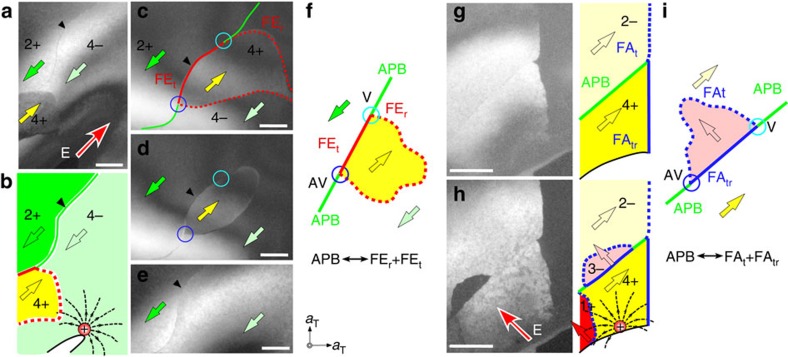
The domain switching kinetics under e^−^ beam-induced poling. In-plane DF-TEM images and schematics of domain structures in a CSTO crystal under different directions of induced electric field indicated by red arrows. Coloured arrows and domains represent polarization directions and FE domain states, respectively. Scale bar, 200 nm. (**a**–**e**) Image sequences showing a Z_3_ vortex-antivortex (V-AV) pair evolution during *in-situ* poling within a single ferroelastic domain. (**a**,**b**) The initial state. (**c**,**d**) The states during the charge dissipation process after defocusing electron beam. (**e**) The final state. With a focused electron beam at the sample edge, a direct 180° polarization reversal is observed (4−→4+) via V-AV pair creation (**a**–**c**), and the created domain disappears slowly with charge dissipation, which accompanies V-AV pair annihilation (**c**–**e**). Cyan and blue circles denote Z_3_ vortices and antivortices, respectively. A black arrowhead is the location marker. (**f**) Schematic showing the 180° ferroelectric polarization switching via splitting or coalescence of a APB into two ferroelectric walls: APB (green line)←→FE_t_ (red-solid)+FE_r_ (red-dotted) DWs. (**g**,**h**) Image sequences and schematics showing 90° ferroelectric domain switching near a APB (green line). (**g**) The initial state. (**h**) The immediate image after electron beam focused at the sample edge away from the FA boundary (solid blue line). Only 90° poled domains (dark contrast) are observed (2−→3− and 4+→1+). The 90° poled domains are assigned with the rotation order parameter same with those of the initial domains, which is consistent with the low-energy nature of FA_t_ DW. The induced 3− (pink) and 1+ (red) domains return slowly to the initial 2− (light yellow) and 4+ (yellow) states with charge dissipation. (**i**) Schematic showing a 90° ferroelectric polarization switching within a FA domain via splitting or coalescence of a APB into two ferroelastic walls: APB ←→ FA_tr_ (blue-solid)+FA_t_ (blue-dotted) DWs. Emphasize that there is no hint of the presence of any intermediate states corresponding to 90°polarization reversal during this process.

*In-situ* poling results on CSTO using a DF-TEM technique unveil intriguing domain switching kinetics, which can be understood in terms of the creation and annihilation of Z_3_ vortex-antivortex (V-VA) pairs. *In-situ* poling is achieved utilizing fast positive charging^40^,^41^ induced by focusing the electron beam (∼300 nm in diameter) of the TEM at a thin and local area, and the slow reduction of effective electric fields, because of charge dissipation, occurs after removing the focused beam. Thus, in order to observe the *in-situ* poling process, DF-TEM images are obtained immediately after defocusing the electron beam, before the charges are completely dissipated (on the order of 30 min). Figure 5a–e shows a Z_3_ V-VA pair (cyan and blue circles) within one FA domain behaving coherently with *in-situ* poling. *In-situ* poling at a 5 o'clock position near the crystal edge induces a direct 180° polarization reversal of a 4− (light green) domain to a 4+ (yellow) domain (Fig. 5c), which is accompanied by the creation of a V-AV pair. The induced 4+ domain shrinks slowly after defocusing the electron beam (from Fig. 5c–e). Eventually, the induced 4+ domain disappears, and at the same time, the V-AV pair annihilates. This result demonstrates a 180° polarization reversal associated with the creation or annihilation of a Z_3_ V-AV pair. Furthermore, the results in Fig. 5a–e reveal that APBs act as nucleation reservoirs for the Z_3_ V-AV pair creation and annihilation (Supplementary Fig. 5 and Supplementary Movie 2 for more details). Figure 5f illustrates that in a Z_3_ V-AV pair creation process, a segment of a APB becomes two FE DWs (one FE_r_ DW and the other FE_t_ DW); FE_t_ DWs with a larger energy than that of FE_r_ tend to be pinned at the original APB location, whereas FE_r_ DWs tend to be mobile. This observation is in accordance with the energy hierarchy of FE_r_≤FE_t_≤APB discussed earlier.

We also studied electron beam-induced poling in different directions. The switching process depends significantly on the electric field orientation (Supplementary Fig. 5). For example, Fig. 5g shows another region with two antiphase 4+ (yellow) and 2− (light yellow) domains with slightly different bright contrasts located next to an APB (green line). Interestingly, when the electron beam is focused on the specimen edge away from FA DWs (blue lines) and an electric field (red/white arrow in Fig. 5h) perpendicular to the original polar axis is induced, a 90° polarization switching from a bright 2− (light yellow) to dark 3− (pink) triangular domain is observed. The induced 3− domain returns slowly to the initial 2− state with charge dissipation. This process is involved with the splitting or coalescence of an APB to two FA DWs; one FA_tr_ DW and the other FA_t_ DW (Fig. 5i). The created FA_tr_ DW with high energy stays at the original APB location, whereas the created FA_t_ DW with low energy tends to be mobile.

## Discussion

**Figure 6:**
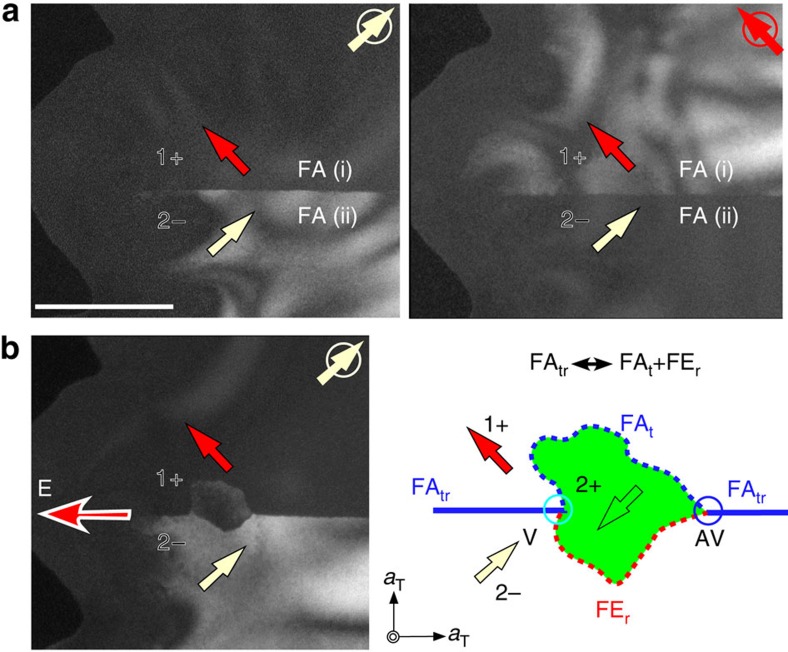
Domain switching kinetics in the absence of APBs in CSTO. (**a**) In-plane DF-TEM images show two ferroelastic (FA(i) and FA(ii)) regions. Domain states 2− (light yellow arrow) and 1+ (red arrow) are assigned based on diffraction patterns and the assumption of a non-charged head-to-tail wall. (**b**) Polarization domain evolutions across a FA_tr_ boundary. Election beam focused at the end of a FA boundary close to the sample edge induces an electric field indicated by a red/white arrow. A polarization switching (dark grey contrast) from 1+ → 2+ in FA(i) and 2−→2+ in FA(ii) is observed. The right-side cartoon shows a schematic of the domain switching via a splitting/merging of FA_tr_ ←→ FA_t_+FE_r_ DWs. A high energy FA_tr_ DW becomes two DWs with lower energies; one ferroelectric FE_r_ DW and the other ferroelastic FA_t_ DW, which is consistent with our energy hierarchy. Both FA_t_ and FE_r_ DWs tend to be mobile and highly curved, which is again consistent the low energy nature of FA_t_ and FE_r_ DWs in our energy hierarchy. Cyan and blue circles denote Z_3_ vortex (V) and antivortex (AV), respectively. Scale bar, 500 nm.

We observe a direct 180° (90°) polarization switching, instead of going through an intermediate 90° (180°) polarization state in the *in-situ* poling process. We emphasize that the coherent DW network of Z_4_ × Z_2_ domains with Z_3_ vortices and some highly curved walls (Fig. 3) leads to the presence of charged DWs and APBs. The presence of Z_3_-vortices instead of the formation of antiparallel domains separated by neutral walls also indicates that a moderate energy difference among five types of DWs. In particular, those APBs serve as primary nucleation centres for 180° and 90° polarization switching, and the presence of mobile *n*-type charge carriers, screening the polar discontinuity at charged DWs, are responsible for the large conduction of head-to-head DWs^3^. Note that the APBs with the discontinuity of both octahedral tilting (t) and rotation (r) costs more energy and one ABB splits into two FE/FA walls under an external electric field, so an APB becomes a nucleation centre of a new poled domain (Fig. 5). This can also happen in high-energy FA_tr_ DWs as shown in the Fig. 6. This zipper-like splitting of high-energy DWs accompanies the emergence of Z_3_ V-AV pairs. The high-energy APB and FA_tr_ DWs dominate the nucleation controlled kinetics of polarization flipping, while the low-energy FE_r_ and FA_t_ DWs tend to move steadily with an external electric field, which can be responsible for the DWs motion kinetics. Emphasize that unlike expected minor roles of APBs with just translation phase shift, APBs dominate the FE polarization switching in hybrid improper FE (Ca,Sr)_3_Ti_2_O_7_. These unexpected discoveries of the role of APBs and the domain topology relevant to the presentence of abundant conducting DWs should be further investigated for deeper understanding and nano-engineering of the domains and DWs in hybrid improper FEs.

## Methods

### Sample preparation

Single-crystalline CSTO and CMTO were grown by using optical floating zone methods. For polycrystalline Ca_3-x_Sr_x_Ti_2_O_7_ (Ca_3_Mn_2-x_Ti_x_O_7_) feed rods, stoichiometric CaCO_3_, SrCO_3_ and TiO_2_ (MnO_2_) were mixed, ground, pelletized and sintered at 1,350–1,550 °C for 30 h. Substituting Sr into the Ca site in Ca_3_Ti_2_O_7_ induces the reduced size of FA domains suitable for TEM studies. It was very difficult to grow high-quality single crystals of pure Ca_3_Mn_2_O_7_, but the slight substitution of Ti into the Mn site of Ca_3_Mn_2_O_7_ stabilizes crystal growth without changing the relevant physics of Ca_3_Mn_2_O_7_. Crystals are highly cleavable, and were cleaved in air for optical microscopy observation. Transparent amber coloured CSTO single crystals and non-transparent dark blue coloured CMTO present a similar polar orthorhombic symmetry (space group #36, *A2_1_am*)^3^,^30^,^33^. Crystal structure and lattice parameters were examined by X-ray diffraction with a Philips XPert powder diffractometer and the general structure analysis system program. CMTO possesses a one and half larger FA distortion (that is, orthorhombicity defined by (*a*−*b*)/(*a*+*b*)*100%, ∼0.08 %) than Ca_3_Ti_2_O_7_ (∼0.05 %). Cycling the CMTO sample temperature through Tc leads to a completely different irregular FA pattern, indicating that the domain formation is not simply due to pinning by disorder such as chemical defects or dislocations.

### Dark-field TEM measurement

**Figure 7:**
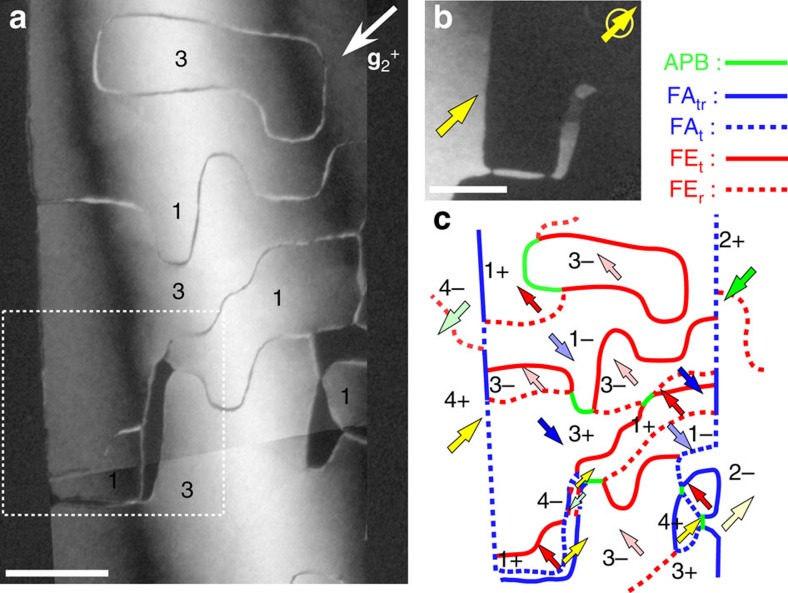
A full assignment of domain states and wall types in CSTO. (**a**) A 2 × 2.9 μm^2^ mosaic of DF-TEM images were taken using the superlattice **g**_**2**_^+^=3/2(−1, 1, 2)_T_ spot of domain FA(i) in a CSTO crystal along [1–11]_T_, showing only part of DWs in this condition. Neither domain contrast nor curved and broad FE_r_ DWs can be visualized under this condition. A tilting (1^st^ or 3^rd^ quadrants) configuration are depicted. Scale bar, 500 nm. (**b**) A DF-TEM image taken using another superlattice **g**_**1**_^**+**^ spot (yellow circle), 90° relative to the one for FA(i), within the white rectangular box. The bright contrast corresponds to the other four types of 180° FE domains (2+, 2−, 4+ and 4−) inside the FA(ii) region. (**c**) A schematic domain and domain wall configuration for the area in **a**, including various FE domains and five types of DWs.

Specimens for DF-TEM studies were fabricated on Ca_3_Ti_2_O_7_ (CTO), Ca_2.55_Sr_0.45_Ti_2_O_7_ (CSTO) and Ca_3_Mn_1.9_Ti_0.1_O_7_ (CMTO) single crystals (∼1 × 2 × 0.1 mm^3^ in size) by mechanical polishing, followed by Ar-ion milling and studied using a JEOL-2010F TEM. Note that one TEM specimen can include up-to-a-hundred FA domains for observations, and we have examined two CSTO, one CTO and two CMTO TEM specimens. Although the width of FA domains varies, our conclusion on the Z_4_ × Z_2_ domain structure with Z_3_ vortex patterns and five types of DWs is universal in all specimens that we have observed. We also found that the FA domain size depends little on various heat treatment conditions in both CSTO and CMTO. We observed vortex domains by DF-TEM imaging taking two diffraction vectors: (i) superlattice **g**_**1**_^±^=±3/2(1, 1, 0)_T_=±(3, 0, 0)_orth_ spots, parallel to the polar axis in [001]_T_ zone and (ii) superlattice **g**_**2**_^±^=±3/2(−1, 1, 2)_T_=±(0, −3, 3)_orth_ spots, perpendicular to the polar axis in [1, −1, 1]_T_ zone, 15° tilting from *c*-axis. Figure 7a shows DF-TEM images taken at the same area as Fig. 3a using the superlattice g_2_^+^ spot. Non-FE structural boundaries can be visualized under this condition as the g_2_^+^ vector is perpendicular to the polar *a*_orth_-axis and the FE contribution is minimized. To avoid confusion with APBs, those boundaries observed in this condition without considering the polarization effect are named as ‘B-boundaries'. The appearance of ‘B-boundaries' is a result of symmetry breaking through the tetragonal-to-orthorhombic phase transition. They tend to show step-like features along the <100>_T_ direction. Based on the domain switching kinetics shown in Fig. 5, we argue that those ‘B-boundaries' are either APBs or FE_t_ DWs, so APBs and FE_t_ DWs tend to be <100>_T_-oriented. Contrarily, FE_r_ DWs show wavy features with no preferred orientation, because of the so-called ‘rotational compatibility conditions'^38^. The role of ‘B-boundaries' is further discussed in Supplementary Fig. 5. Figure 7b shows DF-TEM image taken using the superlattice **g**_**1**_^+^ spot, showing the domain contrast of neighbouring FA(ii) region. A full assignment of domain states and DW types in a CSTO crystal is shown in Fig. 7c based on the domain contrast shown in Figs 3a and 7b and wall features associated with local distortions discussed below.

### Local structural distortions at FE and FA DWs

Supplementary Fig. 2a shows a (110)_T_-oriented FE_t_ DW between two neighbouring 1+ and 3+ domains, where the octahedral tilting (*a*^−^*a*^−^*c*^0^) may be fully suppressed if octahedral tilting changes across the DW by passing through the tetragonal central position (Supplementary Fig. 3). On the other hand, Supplementary Fig. 2b shows a FE_r_ DW between neighbouring 1+ and 1− domains, where neither of the two lattice modes becomes zero. Thus, FE_r_ DW may accompany a lower energy than FE_t_ DW does (Supplementary Fig. 3). Given that the oxygen octahedra in A_3_B_2_O_7_ are connected by sharing the oxygen atom in their corner, at those DWs, some shift of equatorial oxygens (indicated by red spheres in black circles in Supplementary Fig. 2a,b) that locate between Ti sites (cyan spheres) is expected. Enlarged views of the octahedra across the DWs (Supplementary Fig. 2a,b upper panels) clearly show a larger octahedral mismatch at the FE_r_ DW than that at the FE_t_ DW when observed along the [100]_T_ axis. Experimentally, APBs can be identified from the domain contrast without ambiguity (Fig. 2b–d). Although DWs can deviate from typical orientations to minimize the wall energy in the thin foil-type geometry of TEM specimens, we constantly observe a narrow sharp-contrast wall and a relatively broad wall with clear interference fringes near a Z_3_ vortex core. As strain provides the main diffraction contrast change in our DF-TEM images, we associate the narrow sharp-contrast lines with a less octahedral mismatch to FE_t_ DWs in the *ab*-plane projection. This is, indeed, the case for a sharper wall between domain i and iii shown in Fig. 2d and between domains 1− and 3− or domains 1+ and 3+ shown in Fig. 4d,e, which we therefore assign as FE_t_ DWs. A clear interference fringes or wavy features can be observed between domains ii and iii (Fig. 2d) and domains 1− and 1+ or domains 3− and 3+ (Fig. 4d,e), suggesting an inclined nature and a strong strain gradient as expected in FE_r_ DWs.

### Phase-field modeling

To describe the distortion relative to the high symmetry phase with space group *I4/mmm*, three sets of order parameters are used, that is, *ϕ*_*i*_(*i*=3) for the oxygen octahedral rotation around the *x*_3_ axis, and *θ*_*i*_(*i*=1,2) and *P*_*i*_(*i*=1,2) for the octahedral tilt and polarization component along the *x*_*i*_(*i*=1,2) pseudocubic axis, respectively^23^,^42^. The total free energy density can be expressed by


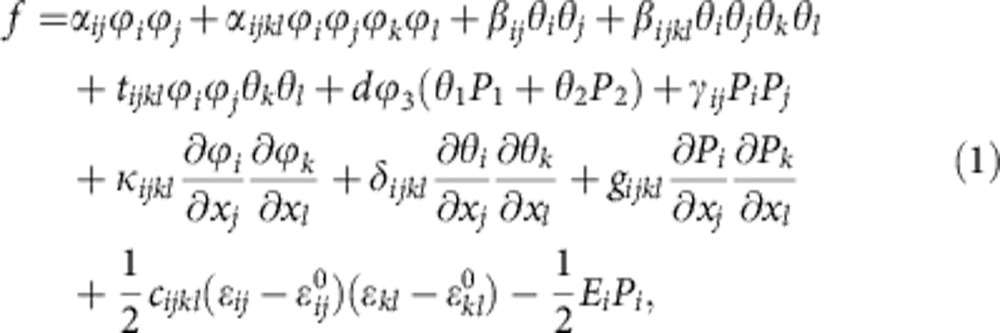


where *α*_*ij*_, *α*_*ijkl*_, *β*_*ij*_, *β*_*ijkl*_, *t*_*ijkl*_, *d* and *γ*_*ij*_ are the coefficients of Landau polynomial, *κ*_*ijkl*_, *δ*_*ijkl*_ and *g*_*ijkl*_ are gradient energy coefficients, *c*_*ijkl*_ is the elastic stiffness tensor, *ɛ*_*ij*_ and 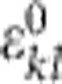 are the total strain and eigen strain, and *E*_*i*_ is the electric field given by *E*_*i*_=−*ϕ*_,*i*_ with *ϕ* the electrostatic potential. Note that *γ*_*ij>0*_, and the term *dϕ*_3_(*θ*_1_*P*_1_+*θ*_2_*P*_2_) determines that (*P*_1_,*P*_2_,0) and (*θ*_1_,*θ*_2_,0) are parallel or antiparallel, dependent on the sign of *ϕ*_3_. The eigen strain is related to the order parameters through 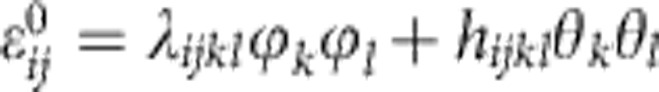, where *λ*_*ijkl*_ and *h*_*ijkl*_ are coupling coefficients. Here the coupling between polarization and strain is ignored, as the secondary order parameter polarization is always parallel or antiparallel to the octahedral tilt order parameter. The Landau polynomial is expanded based on group theory analysis^42^, and the related coefficients are obtained by first-principles calculations^38^,^43^,^44^,^45^,^46^,^47^,^48^ (Tables 1, 2, 3). Anisotropic properties are assumed in gradient energy coefficients *κ*_*ijkl*_ and *δ*_*ijkl*_, that is, *κ*_*iiii*_≪*κ*_*ijij*_, *δ*_*iiii*_≪*δ*_*ijij*_ (ref. 38). The phase-field equations are solved with the initial condition of zero plus a small random noise for the order parameter components^49^. Periodic boundary conditions are employed along the three directions. The system size is 1,024Δ*x* × 1,024Δ*x* × 1Δ*x*, and the grid spacing is Δ*x=*0.30 nm.

### Coefficients of Ca_3_Ti_2_O_7_ used in the phase-field simulations

Total energy calculations based on density functional theory within the generalized-gradient approximation given by the revised Perdew–Becke–Erzenhof parameterization for solids^43^ using the projector augmented wave method^44^,^45^ implemented in the Vienna *Ab initio* Simulation Package^46^,^47^ are used to obtain the coefficients found in the Landau polynomial (Methods, Equation (1)). A plane-wave cutoff of 600 eV and a 4 × 4 × 1 *k*-point mesh with Gaussian smearing (0.10 eV width) is used for the Brillouin-zone integrations. The calcium 3*s*, 3*p* and 4*s* electrons, Ti 3*p*, 3*d* and 4*s* electrons, and O 2*s* and 2*p* electrons are treated as valence states.

The coefficients for the Landau polynomial are obtained by fitting the calculated total energies as a function of magnitude of the order parameters for the configurations corresponding to displacement patterns for each individual mode or combination of modes. The values for the relevant coefficients are given in Table 1. In all total energy calculations, the lattice constants are fixed at the calculated equilibrium values for the high-symmetry *I4/mmm* structure (parent clamping approximation). The total elastic stiffness tensor, including the contributions for distortions with rigid ions and the contributions from relaxed ions, is also obtained by calculating the strain–stress relations^48^ in the *I4/mmm* structure (Table 2). The gradient energy coefficients *κ*_*ijkl*_, *δ*_*ijkl*_ and *g*_*ijkl*_ are estimated based on the gradient energy coefficients of BiFeO_3_ (ref. 38) as both the two systems show the coexistence of oxygen octahedral tilt and polarization, and are listed in Table 3. Note that the domain structures are determined by the relative magnitude of different gradient energy coefficients, and will be hardly affected by the specific values.

### Data availability

The authors declare that all source data supporting the findings of this study are available within the article and the Supplementary Information File.

## Additional information

**How to cite this article:** Huang, F.-T. *et al*. Domain topology and domain switching kinetics in a hybrid improper ferroelectric. *Nat. Commun.* 7:11602 doi: 10.1038/ncomms11602 (2016).

## Supplementary Material

Supplementary Information Supplementary Figures 1-5

Supplementary Movie 1 The nucleation and growth of the ferroelectric A21am phases from the centrosymmetric Acaa matrix in Ca3Mn2O7 are demonstrated by the phase-field simulations. The initial domain structures consist of two variants of the Acaa phase, which are denoted by deep blue and yellow-green colors. The final domains are the eight variants of the A21am phase with the same color assignment as in the main text.

Supplementary Movie 2 A movie of the FEr domain wall motion in a Ca2.55Sr0.45Ti2O7 crystal after defocusing the electron beam of a transmission electron microscope, which corresponds to Fig. 5b to c (the size of the field of view is ∼ 2 μm x 2 μm).

## Figures and Tables

**Table 1:** DFT calculated coefficients for the Landau polynomial.

**Coefficient**	***α***_**33**_	***α***_**3333**_	***β***_**11**_	***β***_**1111**_	***β***_**1122**_	***γ***_**11**_	***t***_**3311**_	***d***
**Unit**	eV f.u.^−1^ Å^−2^	eV f.u.^−1^ Å^−4^	eV f.u.^−1^ Å^−2^	eV f.u.^−1^ Å^−4^	eV f.u.^−1^ Å^−4^	eV f.u.^−1^ Å^−2^	eV f.u.^−1^ Å^−4^	eV f.u.^−1^ Å^−3^
**Value**	−0.3505	0.1868	−0.2024	0.0527	0.0176	0.0188	0.1464	−0.1701

DFT, density functional theory.

**Table 2:** DFT calculated elastic stiffness tensor coefficients.

**Coefficient**	***C***_**11**_	***C***_**12**_	***C***_**13**_	***C***_**33**_	***C***_**44**_	***C***_**66**_
**Value**	315	77.6	98.0	297	81.6	83.1

DFT, density functional theory.

All values are given in units of GPa.

**Table 3:** Normalized gradient energy coefficients.

**Coefficient**	**κ**_**1111**_	**κ**_**1122**_	**κ**_**1122**_	***δ***_**1111**_	***δ***_**1122**_	***δ***_**1212**_	**g**_**1111**_	***g***_**1122**_	***g***_**1212**_
**Value**	0.88	−8.8	8.8	0.88	−8.8	8.8	0.50	−0.088	0.088

All values are normalized with respect to *g*_110_=2.2 eV f.u.^−1^.
