# Supplementary material for: Domain topology and domain switching kinetics in a hybrid improper ferroelectric
Source: Nat Commun. 2016 May 24;7:11602. doi: 10.1038/ncomms11602 (PMC4890185; doi:10.1038/ncomms11602)
Supplement: Supplementary Information — Supplementary Figures 1-5 [file ncomms11602-s1.pdf]

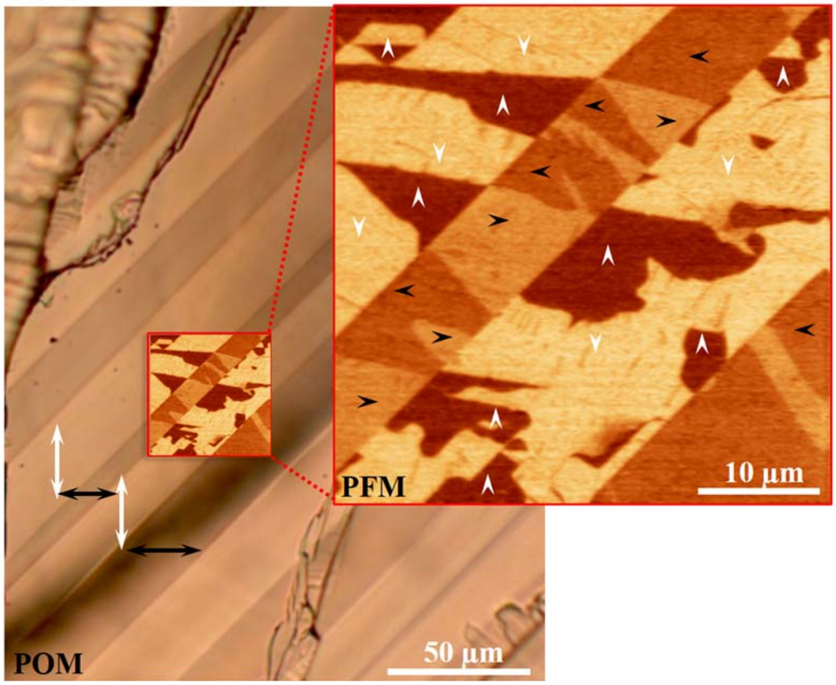

Supplementary Figure 1. In-plane configuration of ferroelastic and ferroelectric domains in $Ca_{2.55}Sr_{0.45}Ti_2O_7$. A polarized optical microscope (POM) image of a cleaved (001) surface at room temperature. The ferroelastic DWs are $[100]_T$-oriented. White and black double arrows indicate the polar *a*-axes in an alternating way. The in-plane piezo-response force microscope (IP-PFM) image of the area in the red box with a scanning area of 40 x 40 μm. The directions of spontaneous in-plane polarization of FE domains are indicated by black and white arrows. There exist four polarization directions along $<110>_T$ axes.

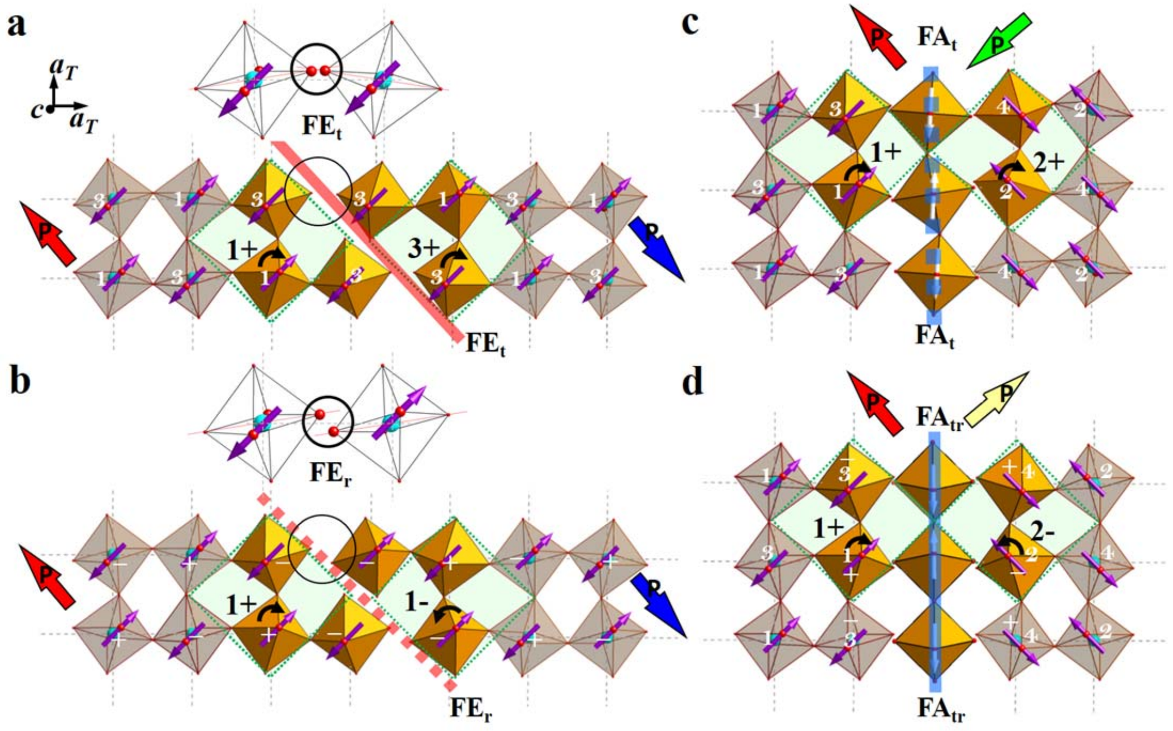

Supplementary Figure 2. Definition of DWs in $A_3B_2O_7$. In-plane illustration of domain walls with BO_6 octahedra represented by orange and gray units. **a,** FE_t DW, red-solid line. A schematic of a $(110)_T$–oriented FE_t DW between two 180°-type FE domains in 1+ and 3+ states. Green dotted lines for the orthorhombic unit cells demonstrate the discontinuation of octahedral tilting ($a^-a^-c^0$, purple arrows) across the wall. White Arabic number 1 or 3 marks the displaced direction of the top apical oxygen in each octahedron. **b,** FE_r DW, red-dotted line. A schematic of a $(110)_T$–oriented FE_r DW between two 180°-type FE domains in 1+ and 1- states. White symbol + (clockwise) or – (counterclockwise) represents the octahedral rotation in each octahedron. **c,** FA_t DW, blue-dotted line. A schematic of a $(100)_T$–oriented FA_t DW between 90°-type FE domains in 1+ and 2+ states. A residual octahedral distortion of either $a^-a^0c^+$ or $a^0a^-c^+$ type at the DW is proposed and shown with white arrows. **d,** FA_{tr} DW, blue-solid line. A schematic of a $(100)_T$–oriented FA_{tr} DW between 90°-type FE domains in 1+ and 2- states. FA_{tr} DWs may adopt a high-symmetry position with single tilt of either $a^-a^0c^0$ or $a^0a^-c^0$ (white arrows) type accompanied by a complete frustration of octahedral rotation at the DW.

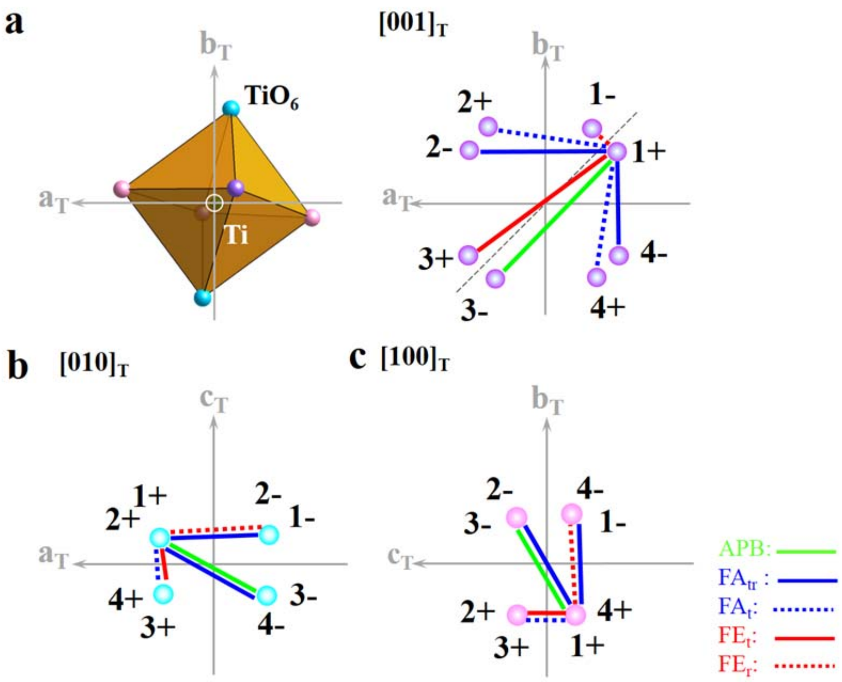

Supplementary Figure 3. Oxygen distortions in eight ferroelectric states. a, A view looking down along the *c*-axis of a TiO_6 octahedron in the 1+ state. In order to reveal octahedral distortions in three dimensions, the oxygen positions in 8 states were projected onto **a,** *ab*-plane, **b,** *ac*-plane and **c,** *bc*-plane. By defining Ti-site (white sphere) as the origin (tetragonal position), off-centered oxygen atoms in eight states are depicted. Purple, cyan and pink spheres represent the apical and two equatorial oxygen pairs, respectively. Spheres depict the oxygen distortions including rotation and tilting. The black dashed line between 1+ and 1- (shown in **a**) illustrates the ideal position when only octahedral tilting exists. The deviation of the 1+ and 1- states from the black line results from octahedral rotation. Eight states (spheres) emerge as a result of octahedral tilting and rotation. The solid and dotted lines indicate various DWs linking different states. For example, the green solid line linking 1+ & 3- represents a APB. FA_t DW; the blue dotted line linking 1+ & 2+. FA_{tr} DW; the blue solid line linking 1+ & 2-. FE_t DW; the red solid line linking 1+ & 3+. FE_r DW; the red dotted line linking 1+ & 1-. By assuming that a wall going through a tetragonal oxygen position costs more energy, we can estimate the energy hierarchy among various DWs in three different planar views: (**a**) $FE_r < FA_t \sim FA_{tr} <$ APB $< FE_t$; (**b**) $FA_t \sim FE_t < FE_r < FA_{tr} <$ APB, and (**c**) $FA_t < FE_t < FE_r < FA_{tr} <$ APB. The averaged DW energy hierarchy appears $FA_t \leq FE_r \leq FE_t < FA_{tr} \leq$ APB. Judging from the displaced length of each DWs, APB and FA_{tr} belong to a higher energy set than others. In addition, the oxygen displacement

can pass through the origin in APBs, FA_{tr} and FE_{t} DWs, implying a complete suppression of structural order parameters the middle of the DWs and thus a non-ferroelectric state in the middle of the DWs.

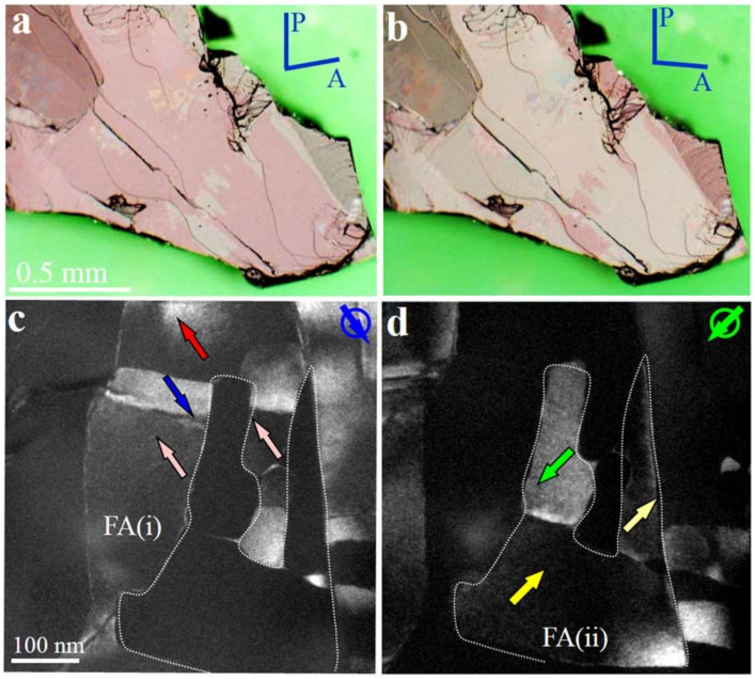

Supplementary Figure 4. Ferroelastic and ferroelectric domains in a CMTO crystal. a-b, The linearly polarized optical microscope (POM) images on the cleaved (001) surface of a $Ca_3Mn_{1.9}Ti_{0.1}O_7$ crystal. The angle between polarizer (P) and analyzer (A) is shown in the figure. Contrast reversal of POM images with the change of analyzer angle and an orthogonal relation of superlattice spots in electron diffraction patterns both confirm those irregular domains as the 90°-oriented FA domains (i.e. orthorhombic twins). **c-d,** DF-images were taken using the g_1^- spot, circled in blue or green in Fig. 3b in the main text. Colored arrows correspond to some of eight FE states, and white dotted lines depict FA DWs between FA(i) and FA(ii).

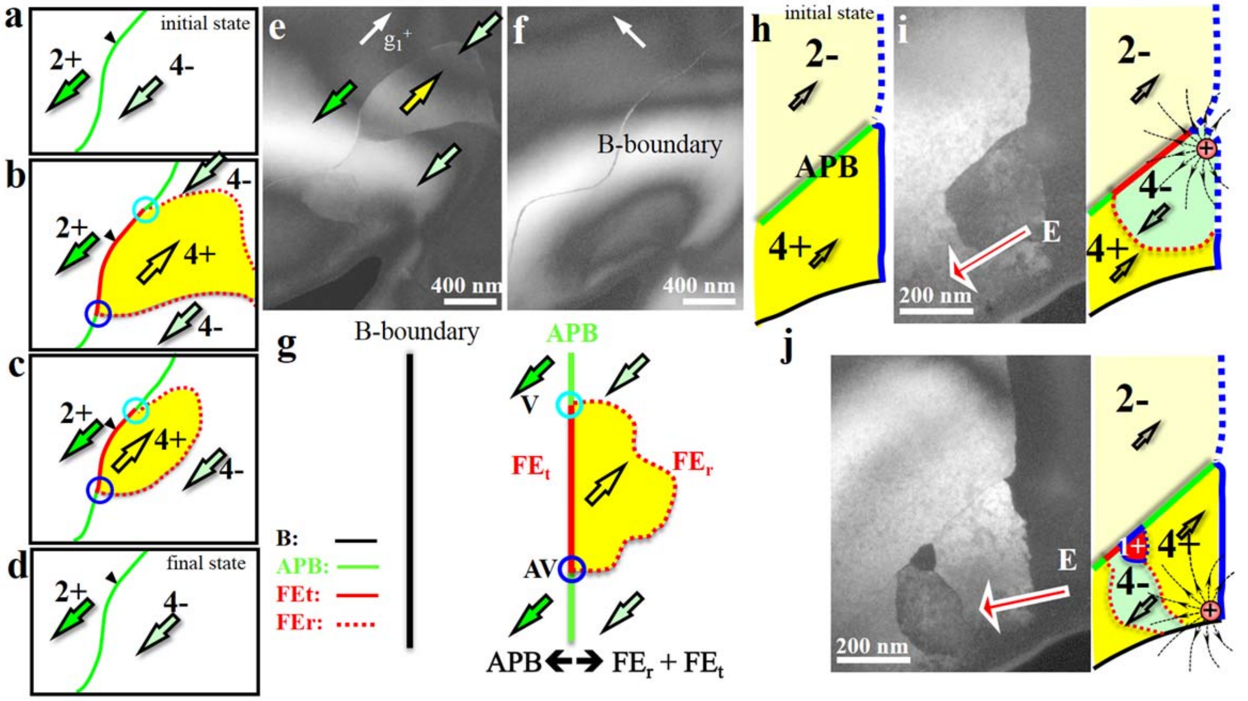

Supplementary Figure 5. Domain switching kinetics close to APBs in a CSTO crystal. a-d, In-plane schematics of beam-induced poling effects correspond to Fig. 5a-d in the main text. Color arrows represent different FE domain states. Cyan and blue circles denote Z_3 vortex (V) and antivortex (AV), respectively. APBs: green solid line; FE_t DWs: red solid line, and FE_r DWs: red dotted line. **e,** A superlattice DF-TEM image taken using the g_1^- spot, corresponding to (**b**). **f,** A superlattice DF-image acquired on the same area as (**e**) using the g_2^+ spot shows a "B-boundary". In this condition, no FE domain contrast is revealed. **g,** A schematic of a DW evolution during a 180° ferroelectric polarization switching via a splitting or coalescence between APB(tr) ←→ FE_t + FE_r DWs. Note that APBs are associated with simultaneous discontinuities of octahedral rotation (r) and tilting (t) at the boundaries, compared with tilting (rotation) discontinuity at FE_t (FE_r) DWs. APB (tr) can split into one FE_t DW and one FE_r DW, and this split accompanies a Z_3 V-AV pair creation as seen from a to b. On the other hand, a V-AV pair annihilation occurs with the coalescence of one FE_t DW and one FE_r DW into one APB as seen from b to d. Our results demonstrate that FE_r DWs can move readily with an applied electric field while FE_t DWs and APBs are attached to "B-boundaries". B-boundaries remain intact during ferroelectric switching, implying that all B-boundaries become APBs in the fully poled state. **h-j,** Electron beam-induced evolutions of FE domains. **h,** The initial state. **i,** With electron beam focused at the center of a FA boundary, a 180° polarization reversal (dark gray

contrast) is observed (4+➔4-). The beam-induced 4- domain returns slowly back to the initial 4+ state with charge dissipation. **j,** Consecutively, electron beam was focused at the bottom of the FA boundary. A major 180° polarization reversal (4+➔4-) and a minor 90° switching (4+➔1+) are observed. Again, the beam-induced domains return slowly back to the initial state. Only 90° poled domains (dark contrast) are observed when the electron beam was consecutively focused at the sample edge away from the FA boundary (Fig. 5g in the main text). Our results demonstrate that the switching process depends significantly on the electric field orientations.
