# Supplementary figures and images for: Domain topology and domain switching kinetics in a hybrid improper ferroelectric
Source: Nat Commun. 2016 May 24;7:11602. doi: 10.1038/ncomms11602 (PMC4890185; doi:10.1038/ncomms11602)

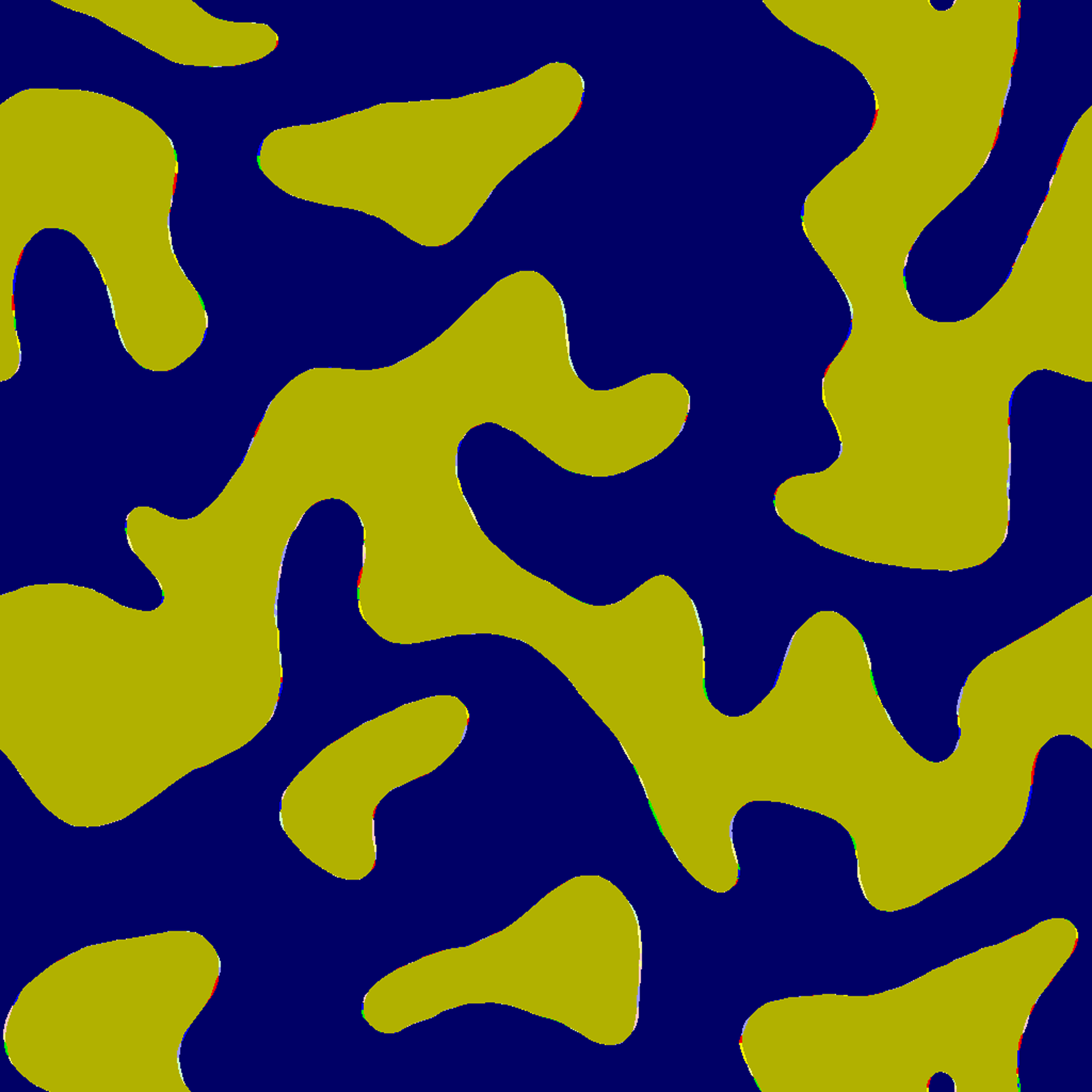

Supplement: Supplementary Movie 1 — The nucleation and growth of the ferroelectric A21am phases from the centrosymmetric Acaa matrix in Ca3Mn2O7 are demonstrated by the phase-field simulations. The initial domain structures consist of two variants of the Acaa phase, which are denoted by deep blue and yellow-green colors. The final domains are the eight variants of the A21am phase with the same color assignment as in the main text. [file ncomms11602-s2.tif]
